# Intersectionality and global health leadership: parity is not enough

**DOI:** 10.1186/s12960-019-0367-3

**Published:** 2019-04-27

**Authors:** Zahra Zeinali, Kui Muraya, Veloshnee Govender, Sassy Molyneux, Rosemary Morgan

**Affiliations:** 1 Department of International Health, Johns Hopkins Bloomberg School of Public Health, 615 N. Wolfe Street, Baltimore, MD 21205 United States of America; 2 Kenya Medical Research Institute (KEMRI) - Wellcome Trust Research Programme, PO Box 43640-00100, Nairobi, Kenya; 3 Department of Reproductive Health and Research, World Health Organization, 20 Avenue Appia, 1211, Geneva 27, Switzerland; 4 Nuffield Department of Medicine, Oxford University, Oxford, United Kingdom

**Keywords:** Gender, Intersectionality, Health systems, Health workforce, Leadership, Global health

## Abstract

There has been a welcome emphasis on gender issues in global health in recent years in the discourse around human resources for health. Although it is estimated that up to 75% of health workers are female (World Health Organization, Global strategy on human resources for health: Workforce 2030, 2016), this gender ratio is not reflected in the top levels of leadership in international or national health systems and global health organizations (Global Health 50/50, The Global Health 50/50 report: how gender responsive are the world’s leading global health organizations, 2018; Clark, Lancet, 391:918–20, 2018). This imbalance has led to a deeper exploration of the role of women in leadership and the barriers they face through initiatives such as the WHO Global Strategy on Human Resources for Health: Workforce 2030, the UN High Level Commission on Health Employment and Economic Growth, the Global Health 50/50 Reports, Women in Global Health, and #LancetWomen. These movements focus on advocating for increasing women’s participation in leadership. While efforts to reduce gender imbalance in global health leadership are critical and gaining momentum, it is imperative that we look beyond parity and recognize that women are a heterogeneous group and that the privileges and disadvantages that hinder and enable women’s career progression cannot be reduced to a shared universal experience, explained only by gender. Hence, we must take into account the ways in which gender intersects with other social identities and stratifiers to create unique experiences of marginalization and disadvantage.

## Main Text

There has been a welcome emphasis on gender issues in global health in recent years in the discourse around human resources for health. Although it is estimated that up to 75% of health workers are female [1], this gender ratio is not reflected in the top levels of leadership in international or national health systems and global health organizations [2, 3]. This imbalance has led to a deeper exploration of the role of women in leadership and the barriers they face through initiatives such as the WHO Global Strategy on Human Resources for Health: Workforce 2030, the UN High Level Commission on Health Employment and Economic Growth, the Global Health 50/50 Reports, Women in Global Health, and #LancetWomen. These movements focus on advocating for increasing women’s participation in leadership. While efforts to reduce gender imbalance in global health leadership are critical and gaining momentum, it is imperative that we look beyond parity and recognize that women are a heterogeneous group and that the privileges and disadvantages that hinder and enable women’s career progression cannot be reduced to a shared universal experience, explained only by gender. Hence, we must take into account the ways in which gender intersects with other social identities and stratifiers to create unique experiences of marginalization and disadvantage. As Horton states: “Gender equality is about more than numerical parity. […] [G]ender is only one dimension of equality. Issues of race and class are also important to consider” [4].

If we are to advocate for equal opportunities in leadership positions in health systems and global health, we must be cognizant of the diverse challenges women face in their daily lives and career advancement in different settings. Gender, a social and inherently political construct, is infused into personal activities and interactions and into organizational structures, practices, and processes, in ways that influence a person’s experience of the world, including their professional development and career advancement. However, it is only one dimension of an individual’s identity and experience. Recognizing the dynamic interconnectedness of gender with other social identities and stratifiers, especially when considering women who do not fit the descriptions of how most women in leadership positions are represented, is integral to developing solutions that benefit all women and to allowing the potential of a truly diverse global health workforce to be tapped into [5].

An intersectional approach “seeks to demonstrate the convergence of different types of exclusion and marginalization” [6]. A concept first coined and used by black legal feminist scholar Kimberle Crenshaw intersectionality moves beyond examining individual factors such as socioeconomic status, sex, gender, race/ethnicity, age, disability/ability, migration status, or religion. Instead, it focuses on the relationships and interactions between such factors, and across multiple levels of society, and how these processes create interdependent forms of privilege and oppression [6, 7]. In other words, intersectionality as an approach helps us to understand and acknowledge the complexity of people’s lives and how different social locations intersect to create unique experiences and positionalities for individuals. An intersectional approach has over the last decade begun to be widely applied in public health, supporting the exploration of the roots of gender inequity in the health sector, how these inequities intersect with other social identities and stratifiers, and identifying opportunities for change [8]. It also promotes a deeper understanding of the dynamic nature of privilege and oppression in permeating health systems and affecting health outcomes [8]. Through supporting an examination of interconnected underlying mechanisms of power [6], it is a valuable framework for explaining and addressing local and global health inequities.

In a recent literature review on intersectional approaches to health systems’ leadership in low and middle-income countries [forthcoming], no study was found that used an explicit intersectionality lens. However, common themes emerging from related studies included important intersections of gender in women’s progress to health leadership positions with race, ethnicity, religion, social networks, and professional cadre [9–11]. The broader literature on women’s leadership in global health also supports that issues of race, caste, age, professional cadre, and class strongly intersect with gender [12]. Given that patriarchal structures manifest in complex, multifaceted and reinforcing ways, the effects of these intersections differ across contexts. The lack of LMIC-based studies using an explicit intersectional lens aimed at exploring the links between experiences of marginalization and disadvantage and broader systems and structures of oppression is an important gap in human resources for health specifically, with implications for the wider governance and health systems discourse.

A better understanding of the ways in which patriarchal and other social and political institutions intersect to affect women of color, indigenous women, women from lower socio-economic backgrounds, women in female-dominated professions such as nursing and midwifery, and academics is needed. Exploring how these institutions of power are infused into organizational structures, processes, and daily life is a priority to inform more equitable policy and practice that tackles and addresses gender and its complex interactions with other social identities and stratifiers [13]. There is strong advocacy to adopt gender-mainstreaming practices and gender-inclusive and transformative policies at international and national levels. We support and advocate for incorporating intersectional approaches as well.

Specifically, we recommend mapping intersectional inequalities at global, national, and sub-national levels in health systems leadership, and incorporating an explicit intersectionality lens in both qualitative and quantitative studies that examine (1) the compounding effects of the intersection of different social identities and stratifiers as barriers and/or enablers of women’s career trajectories and experiences of leadership; (2) how women may be affected by gender bias differently depending on context and their individual social identities; (3) the ways in which experiences of marginalization and disadvantage are political and historical, and how these processes take place within wider structures and systems of oppression; and (4) how organizational policies and practices can minimize discrimination and disadvantage. Only then can we fully appreciate the hidden barriers women from different backgrounds face in their career advancement and begin to effectively address and mitigate them. Concrete policy and practice implications for global health include incorporating intersectional approaches into program planning and implementation to ensure tailoring to the needs of specific sub-populations, and monitoring and evaluation of interventions to identify and include key groups who have been left out [14]. In leadership discourse more specifically, an intersectional approach requires (1) commitment from all levels of health systems to gender equity based on an intersectional approach; (2) adoption and implementation of policies that support and track women’s career progression including mentorship, professional development opportunities, parental leave, flexible family-friendly working arrangements; and (3) commitment to transparently diversifying the workforce at all levels, especially in leadership positions, in a non-tokenistic and meaningful manner.

As Clark has discussed: “making progress in this area [gender equity] is a matter of leadership and political will on the one hand, and deliberate measures and accountability, on the other” [3]. While the world is still behind on achieving global gender parity [15], we should ensure that the parity we are striving towards is inclusive of all identities and stratifiers that intersect with gender.
